# The combination of ropeginterferon alfa-2b with anti-PD1 blockage in adjuvant therapy in HBV-related HCC

**DOI:** 10.1007/s12072-025-10954-9

**Published:** 2025-11-11

**Authors:** Albert Qin, Ming-Chih Ho, Chan-Yen Tsai, Chun-Jen Liu, Pei-Jer Chen

**Affiliations:** 1 Medical Research and Clinical Operations, PharmaEssentia Corporation, Taipei, Taiwan; 2 Department of Surgery, National Taiwan University Hospital, Taipei, Taiwan; 3 Department of Surgery, National Taiwan University Hospital Hsin-Chu Branch, Hsinchu, Taiwan; 4 Department of Internal Medicine, National Taiwan University College of Medicine, Taipei, Taiwan; 5 Hepatitis Research Center, National Taiwan University Hospital, Taipei, Taiwan; 6 Graduate Institute of Clinical Medicine, National Taiwan University College of Medicine, No. 7, Chung Shan South Rd., Taipei, Taiwan

We appreciate the thoughtful commentary on our article [1] by Li et al raised three methodological considerations: the implications of our study cohort’s low-risk profile [2], particularly the absence of microvascular invasion (MVI); the need for direct mechanistic evidence of an antitumor immune response, such as ctDNA assessment; and the possibility that a sequential treatment schedule may be suboptimal for achieving synergy. We address each of these points in detail below.

We agree with Li’s comment that MVI is a paramount predictor of early postoperative recurrence [1]. According to our enrollment criteria, gross vascular invasion confirmed by imaging (CT or MRI) was an exclusion criteria; however, MVI was not excluded [2]. Of all 15 eligible patients, three of them had MVI confirmed by pathologic examinations. All three patients completed the sequential treatment and were alive without cancer recurrence, with one surviving > 3 years and another one for > 5 years. This result suggests that the sequential regimen of ropeginterferon alfa-2b (ropeg) and nivolumab might potentially be effective at suppressing postoperative recurrence in high-risk patients with HBV-related hepatocellular carcinoma (HCC).

It is well established that both HBsAg and circulating tumor DNA (ctDNA) are independent prognostic markers for early-stage HBV-related HCC. Qin et al. [2] previously demonstrated that negative HBV DNA and a short-term decline in quantitative HBsAg (qHBsAg) from baseline are associated with superior survival after PD-1/PD-L1 inhibitor therapy [3]. A decline in qHBsAg, in particular, correlates strongly with favorable prognosis. Based on these findings, we evaluated HBsAg dynamics in our phase 1 study. We fully recognize the importance of ctDNA in assessing antitumor activity and plan to include ctDNA evaluation in the upcoming phase 2 study [4].

Li et al. [1] referenced Zhu’s work to highlight that type I interferons can potentiate responses to PD-1 blockade, partly by upregulating PD-L1 expression on tumor cells [5]. They further proposed that concurrent administration might yield a stronger synergistic effect. We agree that interferon-α and PD-1 blockade can interact synergistically to enhance the efficacy of PD-1 inhibition. However, no evidence currently shows that concurrent administration of type I interferons and PD-1 blockade is more effective than a sequential regimen. As noted by Li et al., type I interferons may augment PD-1 blockade through PD-L1 upregulation. Moreover, type 1 interferons can activate CD8^ + ^ T cell-mediated antitumor response and influence the tumor microenvironment by recruiting cytotoxic CD8⁺ T cells, thereby amplifying anti-PD-1 mediated immune responses [5–7]. These findings support a combination approach with ropeg and anti-PD-1 therapy. Ropeg is a new generation interferon, existing predominantly as a single isomer and can be administered at high dose levels such as 450 ug as used in our study. At this dose level, significant direct anti-cancer cell and indirect immunity-based antitumor effects of ropeg are expected as discussed previously [2, 8]. We agree with Li et al. that concurrent combination of ropeg and anti-PD-1 antibody may be a potential treatment option. However, interferon or anti-PD1 treatment alone can occasionally lead to clinically significant hepatitis in patients with HBV. To minimize potential toxicities and maximize efficacy, we explored using a sequential regimen of ropeg at 450 μg, followed by the anti-PD-1 treatment. Our preclinical data supported that a sequential treatment of rmIFN-α, followed by an anti-mouse PD-1 antibody produced a continuous decline in mean HBsAg levels compared with either agent alone [9]. In addition, sequential therapy can potentially minimize potential toxicities of the simultaneous combinations. Finally, survival results from our phase 1 study show that, after a median follow-up of 1,340 days, all patients remain alive without tumor recurrence except one who prematurely discontinued after the first ropeg dose because of grade 3 anorexia (Fig. 1). This recurrence rate contrasts with prior reports indicating that roughly 50% of HCC patients relapse within 3 years post-surgery if untreated [10].

**Fig. 1:**
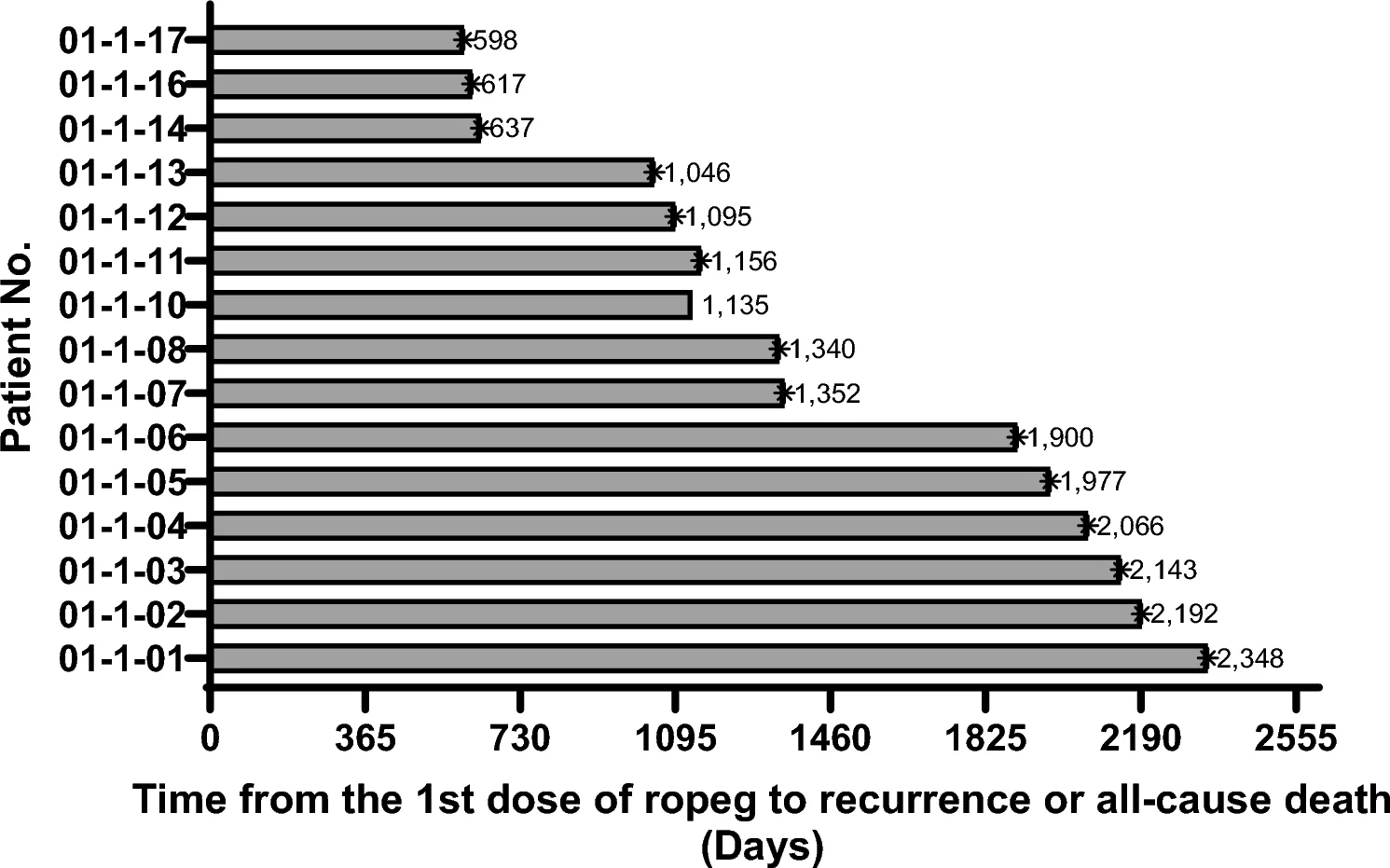
Recurrence-free survival of individual patients receiving sequential therapy with ropeg and anti-PD1 antibody in our study for inhibiting the recurrence of HCC. Patient 01-1-10 experienced recurrence 1135 days after the first ropeg. The other patients are alive without recurrence until the data cutoff date. The symbols of * represent the censor of event

Taken together, our long-term follow-up supports the clinical development of ropeg plus anti-PD-1 as an adjuvant regimen to prevent postoperative HCC recurrence in an upcoming phase 2 trial. While concurrent administration may also exert antitumor effects, sequential treatment has shown promise as an effective regiment in adjuvant therapy in HBV-related HCC.

## Data Availability

The data that support the findings of this study are available from the corresponding author upon reasonable request.
